# m5c-iEnsem: 5-methylcytosine sites identification through ensemble models

**DOI:** 10.1093/bioinformatics/btae722

**Published:** 2024-12-09

**Authors:** Anas Bilal, Fawaz Khaled Alarfaj, Rafaqat Alam Khan, Muhammad Taseer Suleman, Haixia Long

**Affiliations:** College of Information Science and Technology, Hainan Normal University, Haikou 571158, China; Key Laboratory of Data Science and Smart Education, Ministry of Education, Hainan Normal University, Haikou 571158, China; Department of Management Information Systems (MIS), School of Business, King Faisal University (KFU), Al-Ahsa 31982, Saudi Arabia; Department of Software Engineering, Lahore Garrison University, Lahore 54000, Pakistan; Department of Computer Science, Bahria University Lahore Campus, Lahore 54000, Pakistan; College of Information Science and Technology, Hainan Normal University, Haikou 571158, China; Key Laboratory of Data Science and Smart Education, Ministry of Education, Hainan Normal University, Haikou 571158, China

## Abstract

**Motivation:**

5-Methylcytosine (m5c), a modified cytosine base, arises from adding a methyl group at the 5th carbon position. This modification is a prevalent form of post-transcriptional modification (PTM) found in various types of RNA. Traditional laboratory techniques often fail to provide rapid and accurate identification of m5c sites. However, with the growing accessibility of sequence data, expanding computational models offers a more efficient and reliable approach to m5c site detection. This research focused on creating advanced in-silico methods using ensemble learning techniques. The encoded data was processed through ensemble models, including bagging and boosting techniques. These models were then rigorously evaluated through independent testing and 10-fold cross-validation.

**Results:**

Among the models tested, the Bagging ensemble-based predictor, m5C-iEnsem, demonstrated superior performance to existing m5c prediction tools.

**Availability and implementation:**

To further support the research community, m5c-iEnsem has been made available via a user-friendly web server at https://m5c-iensem.streamlit.app/.

## 1 Introduction

**Figure 1.**
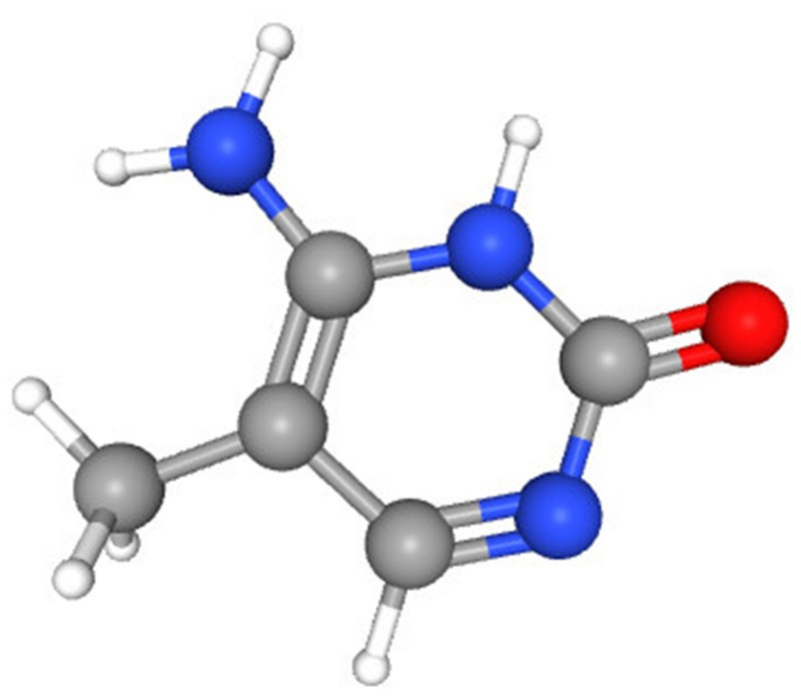
3D structure of m5C.

Post-transcriptional modifications (PTMs) are the changes to RNA molecules after DNA transcription (Suleman *et al.* 2022). These edits can potentially influence both RNA structure and function (El Allali *et al.* 2021). The processing of RNA translation into proteins includes capping, splicing, polyadenylation, methylation, and modifications of base residues that are central to the efficiency of this process (Awazu 2017). More than 150 different PTMs have been discovered, of which 5-methylcytosine (m5c) is the most studied modification (Nombela *et al.* 2021). RNA cytosine 5-methyltransferases and ribonuclease P are enzymes that catalyze a methyl group’s addition to the 5th carbon of the cytosine base on RNA. This particular cytosine methylation greatly influences various biological processes and can alter gene expression, RNA splicing, and mRNA stability. Diseases have been related to the m5c modification, including breast cancer (Yi *et al.* 2017), intellectual disability syndrome (Khan *et al.* 2012), spina bifida samples (Franke *et al.* 2009), Cri du Chat syndrome (Wu *et al.* 2005), and Dubowitz syndrome phenotype samples (Martinez *et al.* 2012). The modification in this manner happens when a methyl bunch (–CH3) is incorporated with the cytosine at the end of the day, as appeared in Fig. 1.

This alteration has a substantial impact on multiple immune cell subtypes, such as dendritic cells (DCs), natural killer (NK) cells, mast cells, macrophages, T and B lymphocytes, or granulocytes, resulting in a complex immune response in the tumor microenvironment (TME) (Gu *et al.* 2023). Research by Chen *et al.* (2022) showed that m5C modification served as an essential regulator of the TME in colorectal cancer. Liu *et al.* (2022) suggested a tool called m5Cpred-XS, which utilizes convolutional neural networks, was developed by Wang *et al.* The method they used in their study was a selection strategy based on BFET and then model training. One of the models applied for *Homo sapiens*, *Mus musculus*, and *Arabidopsis thaliana*, achieved accuracy at 80.4%, 72.3%, and 77.2%, respectively. Li *et al.* (2018) established four machine-learning models for predicting m5C sites within RNA sequences, with the one based on random forest (RF) was chosen to implement RNAm5cfinder. Their dataset includes samples for *H. sapiens* and *M. musculus*, giving AUC values of 0.87 and 0.77 separately for generic m5C sites and tissue-dependent m5C sites [195]. Lv *et al.* trained five computational models (Lv *et al.* 2020) on data from *A. thaliana*, *Saccharomyces cerevisiae*, *M. musculus*, and *H. sapiens*. The feature extraction methods were the same as above and divided into natural vector, pseudo-k-nucleotide composition (PseKNC), K-tuple nucleotide frequency component (KNFC), and mono-nucleotide binary encoding (MNBE). Using these features, this tool constructed an RF-based predictor named iRNA-m5C, which proved to be better than the existing methods. Chen *et al.* described that two, m5Cpred-SVM, on detection of m5C sites in RNA sequences of *A. thaliana* and *M. musculus* (Chen *et al.* 2020). They obtained six features stemming from RNA sequence segments and used a sequential forward feature selection procedure to determine the most informative subset of features. To solve this problem, in this work, we try to improve the identification ability of computational models for m5C sites by designing novel feature extraction strategies. We use publicly available datasets to do a fair comparison between multiple methods and apply them to the real biological sequences (*A. thaliana*, *S. cerevisiae*, *H. Sapiens*, and *M. musculus*). Nucleotide composition, the relative and absolute positioning of frequencies for canonical nucleotides within sequences, as well as frequency of occurrence were incorporated into the models. Independent samples were only available for *A. thaliana*, so only an independent test of this species’ data was conducted. The model was robust as it was validated using a Jackknife testing and k-fold cross-validation. Results showed that m5C-iEnsem was the most accurate among all m5C predictors being maintained at the time of evaluation tests using the same datasets for comparison.

## 2 Materials and methods

### 2.1 Dataset accumulation

**Figure 2.**
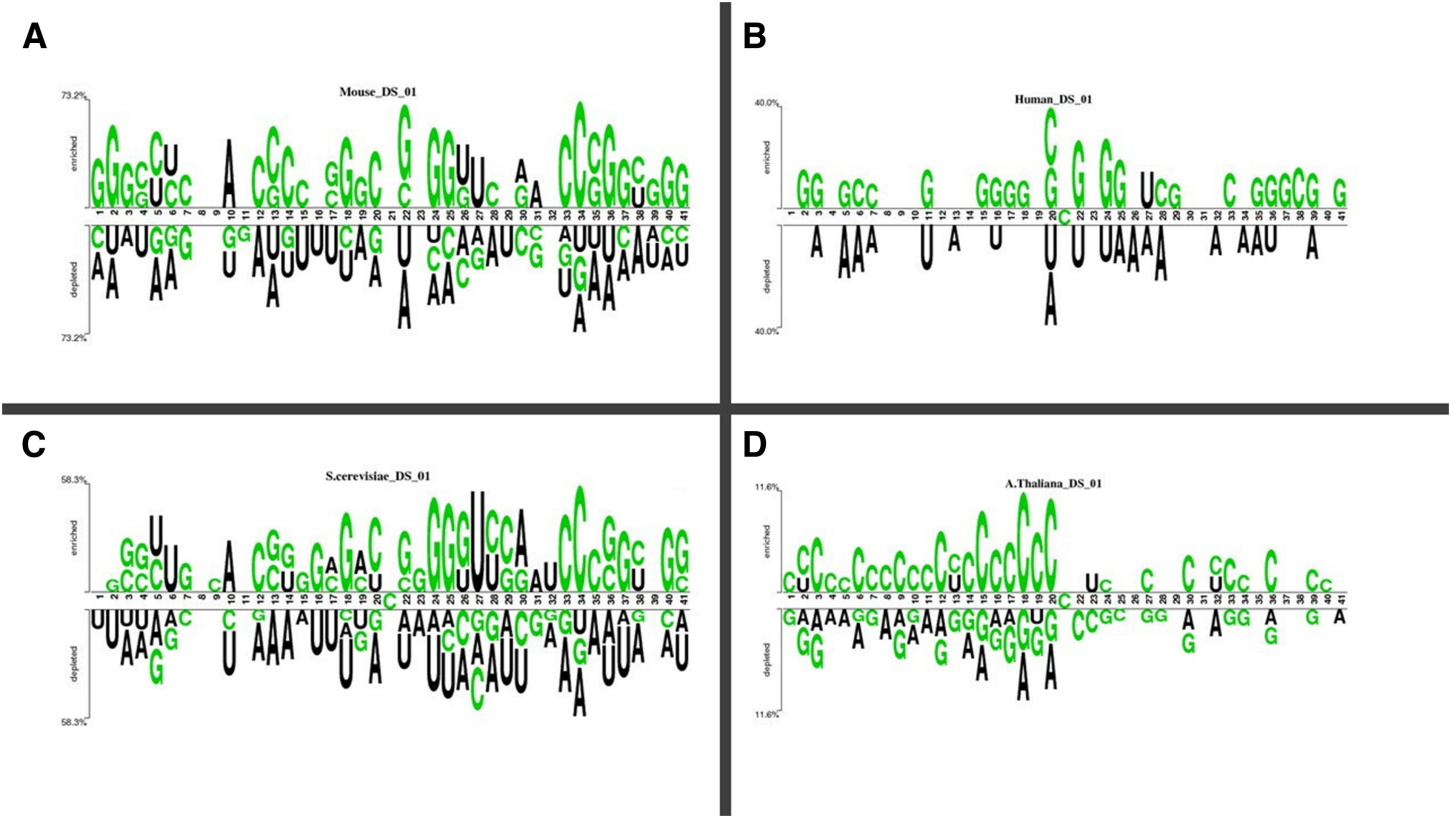
Two sample logos of DS_01. (A) *Mus musculus*. (B) *Homo sapiens*. (C) *Saccharomyces cerevisiae.* (D) *Arabidopsis thaliana*.

**Figure 3.**
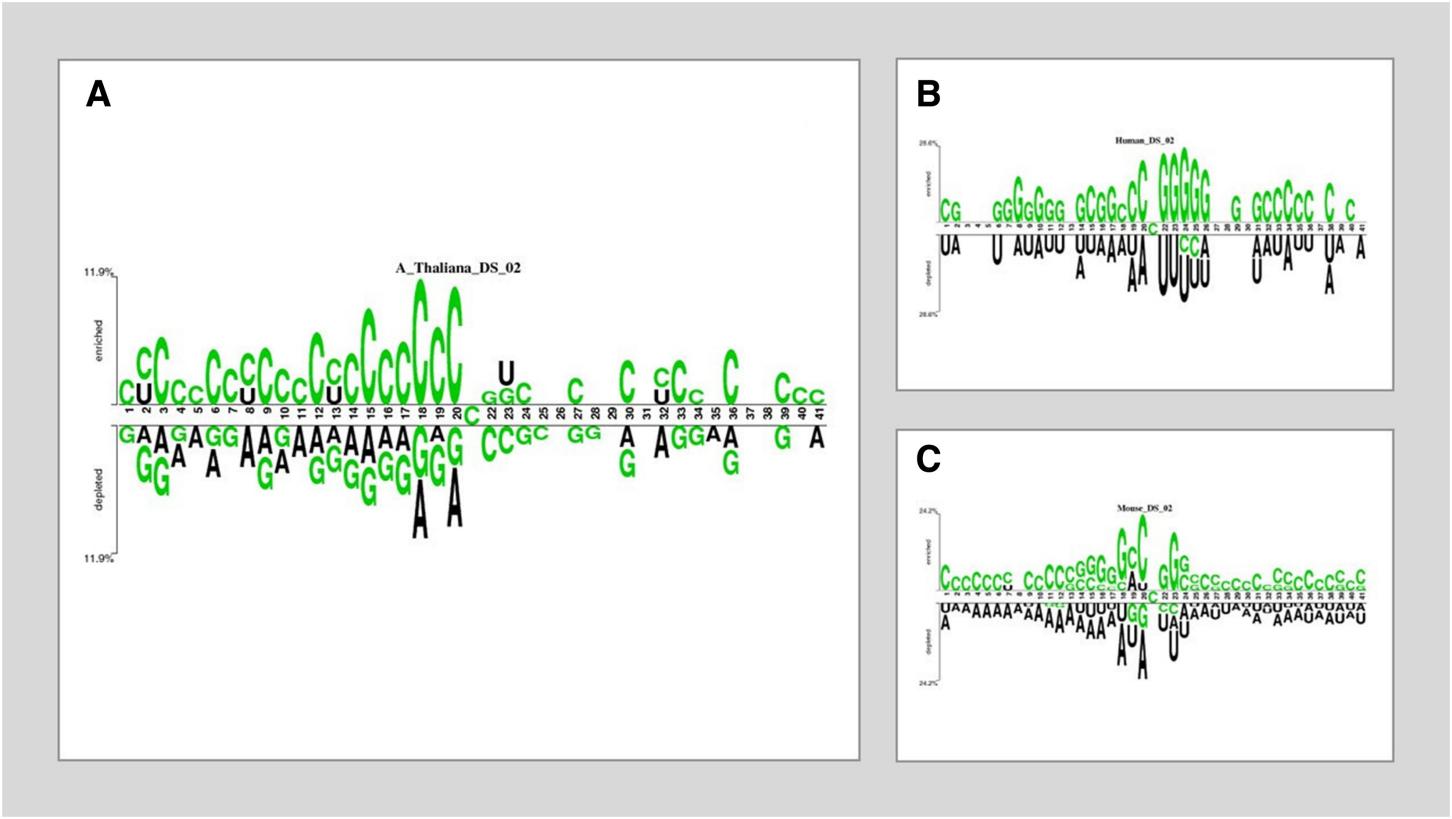
Two sample logos of DS_02. (A) *Arabidopsis thaliana.* (B) *Homo sapiens*. (C) *Mus musculus*.

**Figure 4.**
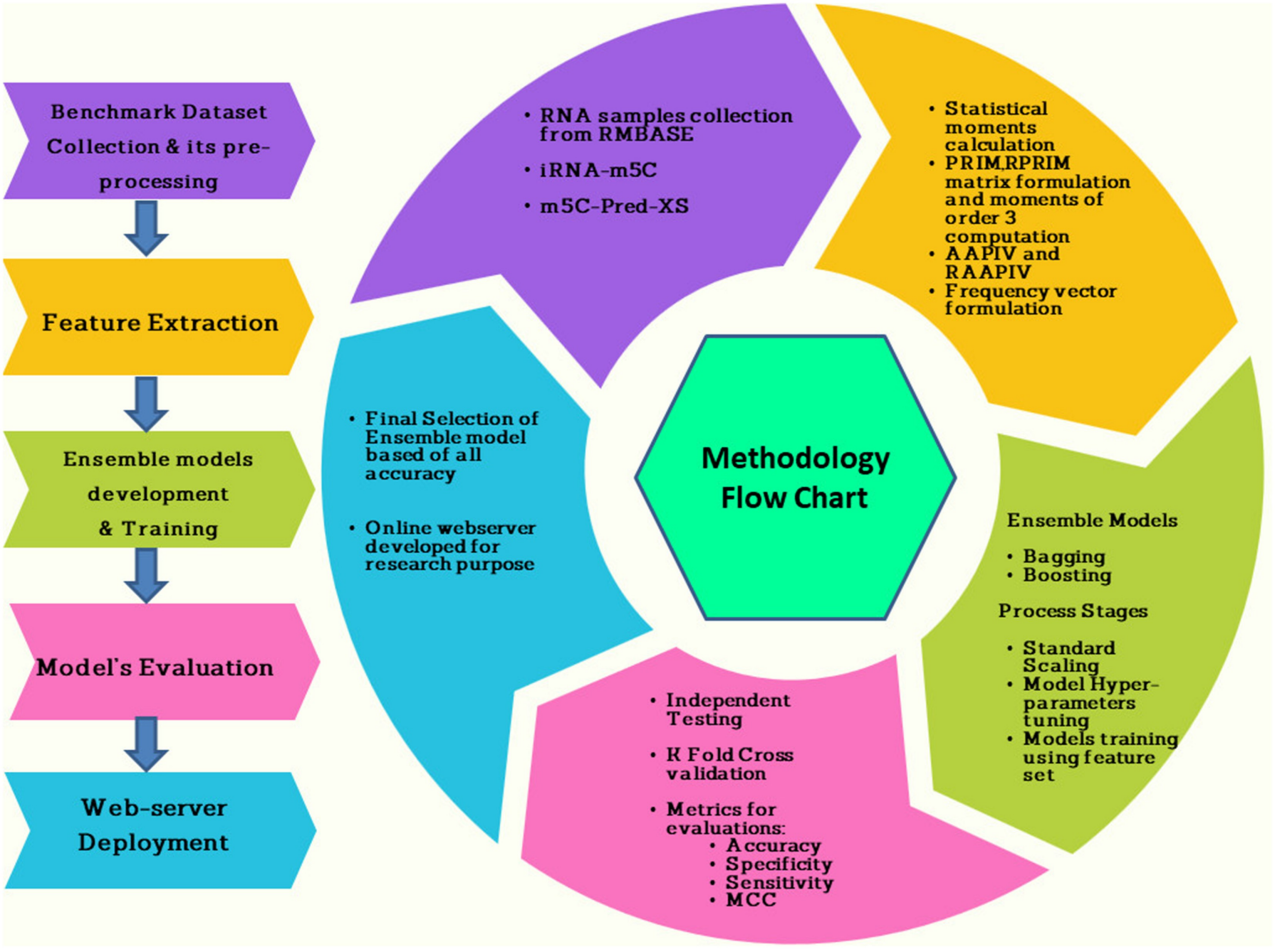
Proposed methodology.

Two sets of datasets were used to train and test the ensemble models in this study. The two datasets (referred to as DS_01 and DS_02 in Table 1) have been used by Lv *et al.* (2020) and Liu *et al.* (2022), respectively. Table 1 species breakdown of the data samples for each dataset DS_01 contains all the *A. thaliana* independent samples but no *S. cerevisiae* samples, whereas DS_02 does not include *S. cerevisiae* samples. The sample compositions of these datasets were selected by diversity. In this context, true positive samples are m5C sites within an RNA sequence, while true negatives would be non-m5C sites. DS_01 is composed of 5717 positive and 5717 negative samples; the same goes for DS_02, serving 12 121 positive and again 12 121 negative samples showing that they are balanced datasets. The RNA samples included in this study are of fixed length; each sample is 41 bp long. The samples from the AraID single-species dataset DS_01 are visualized as a two-sample logo (Vacic *et al.* 2006) in Fig. 2, the DS_02 logos appear in Fig. 3. The work was done in different stages and those are benchmark datasets identification, setting of samples, feature extraction and Modeling training/testing. Furthermore, we established a publicly available server that can be effectively used for Ψ site detection. As shown in Fig. 4, the study’s process has five central methodological steps.

**Table 1.** Dataset details for training and testing Ensemble models.

Dataset	*Mus musculus*	*Arabidopsis thaliana*	*Homo sapiens*	*Saccharomyces cerevisiae*
DS_01	194 (97 positive, 97 negative)	10 578 (5289 positive, 5289 negative)	240 (120 positive, 120 negative)	422 (211 positive, 211 negative)
Independent Dataset	N/A	2000 (1000 positive, 1000 negative)	N/A	N/A
DS_02	11 126 (5563 positive, 5563 negative)	12 578 (6289 positive, 6289 negative)	538 (269 positive, 269 negative)	N/A

### 2.2 Features extraction

Mapping RNA sequences as a feature vector is rather widespread, as the computational models cannot directly work with biological sequences. By statistically analyzing collected samples, it becomes feasible to uncover hidden information within these sequences (Ali *et al.* 2023) (Ahmed *et al.* 2022). In this study, particular emphasis was placed on the feature generation process, which relied on both position and nucleotide composition in a sequence. To address the loss of sequence patterns in protein sequences, Chou recommended the use of pseudo amino acid composition (PseAAC), which is widely acknowledged as one of the most common and effective methods (Arif *et al.* 2022, Nour *et al.* 2022). Following the strategy outlined in PseAAC, the current research adopted a similar approach to construct the feature vector. Within the scope of this research, the feature vectors are meticulously crafted to highlight both the position and nucleotide content within the sequence (Shah and Khan 2020, Shah *et al.* 2022). The description of samples in the dataset was based on the nucleotide formulation as referenced in (1).

(1) $$P_{\epsilon }\left(K\right)={\left[{£}_{1}{£}_{2}{£}_{3}\;\ldots {£}_{U}\;\ldots {£}_{\Omega }\right]}^{T}$$

This, in terms of the *K*-tuple nucleotide scheme used in the study, the constituent vectors are determined based on the feature generation model utilized. These are expressed symbolically: *T* is a symbol for transposing the compiled feature formulation within this process. The effective sequencing length for each nucleotide at that site was 41 bp as identified by (2).

(2) $$P=R_{1}R_{2}R_{3}\cdots R_{18}R_{19}R_{21}\cdots R_{39}R_{40}R_{41}$$

In Equation (3),$\;R_{21}=U\;$ and all the values of$\;R_{n}\;$(where *n* = 1, 2………, 41; *n* ≠ 21) stand for any nucleotide containing guanine, adenine, cytosine or uracil.

#### 2.2.1 Statistical moment incorporation

The quantity of each nucleotide collected sequence is contingent upon the composition and position of the nucleic acid sample sequences. Statisticians and data analysts have endeavored to extend moments to various types of data distributions (Suleman and Khan 2022, Suleman *et al.* 2023). This study utilized first, second, and third-order raw, central, and Hahn moments as features during feature extraction phase (Hussain *et al.* 2019). Discrete and background textures depend on scale and place, while balance and proximity traits depend on scale and nearby. Therefore, descriptive statistics used simple graphical summaries that involved raw and central moments to document the likelihood of observing a mean, the asymmetry of the sample data and calculated variances for the resultant dataset. Conversely, Hahn moments were derived using Hahn polynomials to preserve sequence order information (Butt and Khan 2020). These moments were used as a feature extraction method, as demonstrated by (Butt *et al.* 2022), facilitating the identification of membrane proteins. Matrix (3) represents an m*n 2D matrix of nucleotide bases in sequences, with each matrix component denoted by Kʹ.

(3) $$Kʹ=\left[\begin{array}{cccc} k_{11} & k_{12} & \ldots & k_{1n}\\ k_{21} & k_{22} & \ldots & k_{2n}\\ \vdots & \vdots & \ddots & \vdots \\ k_{m1} & k_{m2} & \ldots & k_{mn} \end{array}\right]$$

To extract location variant features from the dataset, moments of the first, second, and the probability distribution were computed, along with the values of the features, means, and variances of these features. The probability distribution function with unequal probability weights was also determined. These scatter variables were derived from the raw moments outlined in (4), in which the total of raw moments represented by terms such as$\;R_{00}$,$\;R_{01}$,$\;R_{10}$,$\;R_{11}$,$\;R_{12}$,$\;R_{21}$,$\;R_{30}$, and$\;R_{03}$, which are polynomials up to the third degree.

(4) $$R_{uv}=\sum_{a=1}^{m}\sum_{b=1}^{m}a^{u}b^{v}\beta_{ab}$$

Central moments are independent of location and instead pertain to the composition and form of distribution (Lo and Don 1989, Khan *et al.* 2014). These moments are determined by the discrepancies between the mean and the random variable (Malebary *et al.* 2019). In this investigation, we calculated the central moments using the expression provided in (5).

(5) $$n_{ij}=\sum_{b=1}^{n}\sum_{q=1}^{n}{\left(b-x\right)}^{i}{\left(q-y\right)}^{j}\beta_{bq}$$

Hahn moments (Zhou *et al.* 2005) were calculated using Hahn polynomials, as outlined in (6).

(6) $$\begin{array}{c} h_{n}^{u,\;v}\left(r,\;N\right)={\left(N+V-1\right)}_{n}{\left(N-1\right)}_{n\;}\\ \times \sum_{k=0}^{n}{\left(-1\right)}^{k}\frac{{\left(-n\right)}_{k}{\left(-r\right)}_{k}{\left(2N+u+v-n-1\right)}_{k}}{{\left(N+v-1\right)}_{k}{\left(N-1\right)}_{k}}\frac{1}{k!} \end{array}$$

To ascertain the orthogonal normalized Hahn moments of the 2D data, we used the equation specified in (7).

(7) $$H_{ij}=\sum_{q=0}^{N-1}\sum_{p=0}^{N-1}\beta_{ij}h_{j}^{\tilde{u,\;v}}\left(q,N\right)h_{j}^{\tilde{u,\;v}}\left(p,N\right),m,n=0,1,\ldots N-1$$

#### 2.2.2 PRIM construction: positional incidence matrix

The current study can, therefore, be considered as the continuation of the above study, given that it sought to conduct further improvements on the performance of the existing model as far as its prediction capacity was concerned. However, for the abovementioned purpose, it was possible to design a model of feature generation using available RNA sequences. The positions of nucleotides in the RNA strand make practical sense and can be used to reference mathematically defined expressions. In order to validate this proposition, the following three relative incidence matrices were constructed: the first one relates to single nucleotide composition (SNC); the second one relates to di-nucleotide composition (DNC); the last one involves tri-nucleotide composition (TNC). These matrices were later used to identify the positional relation of the different nucleotide bases to properly quantify the positional relation of the nucleotides. The matrix,$\;A_{\mathrm{prim}}$, which form a 4 by 4 matrix, whereby 16 coefficients derived from this matrix are expressed in (8).

(8) $$A_{\mathrm{prim}}=\left[\begin{array}{cccc} {Ԏ}_{A\to A} & {Ԏ}_{A\to G} & {Ԏ}_{A\to U} & {Ԏ}_{A\to C}\\ {Ԏ}_{G\to A} & {Ԏ}_{G\to G} & {Ԏ}_{G\to U} & {Ԏ}_{G\to C}\\ {Ԏ}_{U\to A} & {Ԏ}_{U\to G} & {Ԏ}_{U\to U} & {Ԏ}_{U\to C}\\ {Ԏ}_{C\to A} & {Ԏ}_{C\to G} & {Ԏ}_{C\to U} & {Ԏ}_{C\to C} \end{array}\right]$$

In case of any nucleotide’s positional relationships, other tRNA proteins convey amino acids with the corresponding anti-codon nucleotides, either A, G, U, or C, to the appropriate nucleotide. The$\;B_{\mathrm{Prim}}\;$is a 16*16 matrix (10) represented by DNC, which generates 16 nucleotide variants. We obtained 256 coefficients from this matrix as depicted in matrix (9). If a similar assessment of individual statistical moments were summarized into one value, there would only be 30 coefficients.

(9) $$B_{\mathrm{prim}}=\left[\begin{array}{ccccccc} {ժ}_{AA\to AA} & {ժ}_{AA\to AG} & {ժ}_{AA\to AU} & \ldots & {ժ}_{AA\to j} & \ldots & {ժ}_{AA\to CC}\\ {ժ}_{AG\to AA} & {ժ}_{AG\to AG} & {ժ}_{AG\to AU} & \ldots & {ժ}_{AG\to j} & \ldots & {ժ}_{AG\to CC}\\ {ժ}_{AU\to AA} & {ժ}_{AU\to AG} & {ժ}_{AU\to AU} & \ldots & {ժ}_{AU\to j} & \ldots & {ժ}_{AU\to CC}\\ \vdots & \vdots & \vdots & & \vdots & \vdots & \vdots \\ {ժ}_{GA\to AA} & {ժ}_{GA\to AG} & {ժ}_{GA\to AU} & \ldots & {ժ}_{GA\to j} & \ldots & {ժ}_{GA\to CC}\\ \vdots & \vdots & \vdots & & \vdots & \vdots & \vdots \\ {ժ}_{N\to AA} & {ժ}_{N\to AG} & {ժ}_{N\to AU} & \ldots & {ժ}_{N\to j} & \ldots & {ժ}_{N\to CC} \end{array}\right]$$

The matrix$\;C_{\mathrm{Prim}}$, defined in this study as the matrix of dimensions 64 * 64, consolidates 4096 unique tri-nucleotide sequences. However, by using the central, raw, and Hahn moments in the preceding calculations, 30 coefficients are determined. Therefore, the$\;C_{\mathrm{Prim}}\;$matrix is of 64*64 (10), which incorporates 64 diverse tri-nucleotide sequences ranging from AAA to CCG, CCU, CCC, and out of 4096 coefficients, a total of seven coefficients from 30 can only comprise the integrated central and raw as well as Hahn moments.

(10) $$C_{\mathrm{prim}}=\left[\begin{array}{ccccccc} {ѱ}_{\mathrm{AAA}\to \mathrm{AAA}} & {ѱ}_{\mathrm{AAA}\to \mathrm{AAG}} & {ѱ}_{\mathrm{AAA}\to \mathrm{AAU}} & \ldots & {ѱ}_{\mathrm{AAA}\to j} & \ldots & {ѱ}_{\mathrm{AAA}\to \mathrm{CCC}}\\ {ѱ}_{\mathrm{AAG}\to \mathrm{AAA}} & {ѱ}_{\mathrm{AAG}\to \mathrm{AAG}} & {ѱ}_{\mathrm{AAG}\to \mathrm{AAU}} & \ldots & {ѱ}_{\mathrm{AAG}\to j} & \ldots & {ѱ}_{\mathrm{AAG}\to \mathrm{CCC}}\\ {ѱ}_{\mathrm{AAU}\to \mathrm{AAA}} & {ѱ}_{\mathrm{AAU}\to \mathrm{AAG}} & {ѱ}_{\mathrm{AAU}\to \mathrm{AAU}} & \ldots & {ѱ}_{\mathrm{AAU}\to j} & \ldots & {ѱ}_{\mathrm{AAU}\to \mathrm{CCC}}\\ \vdots & \vdots & \vdots & & \vdots & & \vdots \\ {ѱ}_{\mathrm{AAC}\to \mathrm{AAA}} & {ѱ}_{\mathrm{AAC}\to \mathrm{AAG}} & {ѱ}_{\mathrm{AAC}\to \mathrm{AAU}} & \ldots & {ѱ}_{\mathrm{AAC}\to j} & \ldots & {ѱ}_{\mathrm{AAC}\to \mathrm{CCC}}\\ \vdots & \vdots & \vdots & & \vdots & & \vdots \\ {ѱ}_{N\to \mathrm{AAA}} & {ѱ}_{N\to \mathrm{AAG}} & {ѱ}_{N\to \mathrm{AAU}} & \ldots & {ѱ}_{N\to j} & \ldots & {ѱ}_{N\to \mathrm{CCC}} \end{array}\right]$$

#### 2.2.3 Reverse position relative indices matrix

Feature vector development is the process that can help to build a robust and accurate prediction model (Alghamdi *et al.* 2022). Suppose the numbers are added so that all the numbers in a matrix are placed in the reverse position relative to the original position. The resultant matrix is defined as a Reverse Position Relative Indices Matrix (RPRIM). Therefore, equation (11) can be written as V_RPRIM, where every constituent of vector R_(i→j) relates to the relative positional value about one with nucleotide base vis the jth nucleotide similarly, while estimating RPRIM, as for PRIM matrices, mononucleotide, di-nucleotide, and tri-nucleotide run context were used.

(11) $$V_{\mathrm{RPRIM}}=\left[\begin{array}{ccccccc} V_{1\to 1} & V_{1\to 2} & V_{1\to 3} & \ldots & V_{1\to y} & \ldots & V_{1\to j}\\ V_{2\to 1} & V_{2\to 2} & V_{2\to 3} & \ldots & V_{2\to y} & \ldots & V_{2\to j}\\ V_{3\to 1} & V_{3\to 2} & V_{3\to 3} & \ldots & V_{3\to y} & \ldots & V_{3\to j}\\ \vdots & \vdots & \vdots & & \vdots & & \vdots \\ V_{x\to 1} & V_{x\to 2} & V_{x\to 3} & \ldots & V_{x\to y} & \ldots & V_{4\to j}\\ \vdots & \vdots & \vdots & & \vdots & & \vdots \\ V_{N\to 1} & V_{N\to 2} & V_{N\to 3} & \ldots & V_{N\to y} & \ldots & V_{N\to j} \end{array}\right]$$

#### 2.2.4 Frequency matrices (FMs) generation

Generating attributes need information extraction to determine the location of this sequence and its composition. The frequency vector represents the count for each nucleotide in (12).

(12) $$Ġ=\left\{\delta_{1},\;\delta_{2},\;\ldots .,\;\delta_{n}\right\}$$

The symbol represents the count of each *i*th nucleotide in a sequence$\;\delta_{i}$. The frequency of single nucleotides and pairs of nucleotides has also been determined.

#### 2.2.5 Generation of AAPIV

The inadequacy with the extraction of the compositional information to offer insights into the positions that contributed to each of the nucleotide calculations. To address this problem, different absolute position incidence vectors (AAPIVs) of length 4, 16, and 64 were calculated and denoted by$\;K_{\mathrm{AAPIV}4}\;$(13),$\;K_{\mathrm{AAPIV}16}\;$(14),$\;K_{\mathrm{AAPIV}64}\;$(15).

(13) $$K_{\mathrm{AAPIV}4}=\left\{\rho_{1,}\rho_{2,}\rho_{3,}\rho_{4,}\right\}$$

 

(14) $$K_{\mathrm{AAPIV}16}=\left\{\rho_{1,}\rho_{2,}\rho_{3,}\;\ldots ,\;\rho_{15,}\rho_{16,}\right\}$$

 

(15) $$K_{\mathrm{AAPIV}64}=\left\{\rho_{1,}\rho_{2,}\rho_{3,}\;\ldots ,\;\rho_{63,}\rho_{64}\right\}$$

Any element$\;\rho_{i}\;$is computed as follows:

(16) $$\rho_{i}=\sum_{k=1}^{n}{р}_{k}$$

#### 2.2.6 RAAPIV generation

The scheme used to obtain the above sequence has been named RAAPIV, which assisted in exposure of information that might have been masked in case of the position of nucleotides inside the sequence. Reverse accumulative absolute position incidence vector (RAAPIV) was found to have three sections, which are the RAAPIV of length 4, 16, and 64 amino acids depicted by equations$\;K_{\mathrm{RAAPIV}4}\;$(17),$\;K_{\mathrm{RAAPIV}16}\;$(18), and$\;K_{\mathrm{RAAPIV}64}\;$(19), respectively, but in reverse sequence order.

(17) $$K_{\mathrm{RAAPIV}4}=\left\{\tau_{1,\;}\tau_{2,}\tau_{3,}\tau_{4}\right\}$$

 

(18) $$K_{\mathrm{RAAPIV}16}=\left\{\tau_{1,}\tau_{2,}\tau_{3,}\ldots ,\;\tau_{16}\right\}$$

 

(19) $$K_{\mathrm{RAAPIV}64}=\left\{\tau_{1,}\tau_{2,}\tau_{3,}\ldots ,\;\tau_{64}\right\}$$

#### 2.2.7 Feature vector formulation

Finally, features were extracted by consolidating them into a single feature vector, constituting the finalized set for model prediction. Hence, subsequent steps were implemented to develop an ultimate feature set: (i) the first step of analysis involved the calculation of PRIM and its refined version, RPRIM to reduce the feature dimensionality. (ii) the obtained features have been integrated into the FV, AAPIV, and RAAPIV systems. Finally, we derived a feature vector containing as many as 522 attributes. What should be remembered is that each feature vector refers only to one type of sample present in the dataset. In the case of binary classification, the positive classes were given the label of 1 while the negative classes were given the label of 0.

### 2.3 Ensemble models development and training

There has been great optimism in ensemble learning techniques in solving complex problems in machine learning due to the technique’s effectiveness over the single model approach. It takes advantage of the multiple model’s strengths and can be divided into parallel and sequential methods. The ensemble models can solve real-world issues in ways such as increasing trust, combining the models, predicting several patterns, and using features-based analysis.

Following synchronous ensemble methods like bootstrapping or bagging refer to training many models simultaneously with different training datasets. On the other hand, the sequential methods imply that models are trained sequentially with subsequent learning being performed by the model’s error. Classification using the ensemble was explained in numerous works.

#### 2.3.1 Bagging ensemble

This study involves bagging ensemble methods using techniques such as subsampling with replacement and row sampling to generate diverse subsets of training samples for the base models. Creating these subsets trains each base model on a distinct data section, ensuring that the base models differ. This strategy effectively reduces the variance of the ensemble and enhances its overall performance (Gupta *et al.* 2022). For the current research, random forest (RF), bagging ensemble (BE), extra tree classifier (ETC), and decision tree (DT) have been used for bagging ensemble models.

#### 2.3.2 Boosting ensemble

Boosting improves model performance by sequentially training models to correct the errors of previous ones, thus enhancing the ensemble’s accuracy. It is sequential in nature, where each model focuses on minimizing and rectifying the error made by the preceding model. This approach focuses on the fact that integrating several weak learners with downtrodden individual abilities improves the ensemble’s performance. Different boosting ensemble methods used in the present work include gradient boosting (GB), histogram-based gradient boosting (HGB), Ada-Boost (AB), and extreme gradient boosting (XGB).

## 3 Results

### 3.1 Performance evaluation matrices

Four measures, namely,$\;S_{n}$,$\;S_{p}$, Acc, and Matthews Correlation Coefficient (MCC) have been used for the evaluation of the prediction models that were developed (Kotowski *et al.* 2023, Zhou *et al.* 2023). Accuracy is a commonly used measure of success in clinical models in classification problems and the number of correct checks divided by the total number of checks. This percentage level identifies how accurate the data prediction was for the whole population. Based on the TPR and the FPR especially$\;(S_{p})\;$have focused more on the accomplishment of a binary classification model particularly if the negative class is larger. This determines the range of identifying actual negative cases among all negative incompetent patients as *TN*/(*TN* + *FP*). Sensitivity$\;(S_{n})\;$assesses the results of binary classifier model particularly when the positive class is extremely critical. It describes the extent to which actually positive factors are measured or how many positive values are measured out of the total positive values. It is named as MCC or Matthews Correlation Coefficient, is used in binary classification models especially when the classes are imbalanced. However, MCC uses the TP and the TN, as well as FP and FN concerning the measure of the model. The formulae of these accuracy metrics are given in (20).

(20) Sn=TPTP+FN 0≤Sn≤1Sp=TNTN+FP 0≤Sp≤1Acc=TP+TN/(TP+FP+FN+TN) 0≤Acc≤1 MCC=TP*TN-FP*FN/(TP+FP)(TP+FN)(TN+FP)(TN+FN)-1≤MCC≤1

In this regard, TP stands for the genuine m5C sites while TN is the non m5C sites. The term *FN* means the number of actual m5C sites that are wrongly localized and the term *FP* refers to non-m5C sites that are wrongly localized into m5C sites. One needs to comprehend that these metrics apply exclusively to the systems managing only one class. In examining the performance of the developed system, *FP* and *FN* rates are critical measurements. The present inaccuracy can result to wrong m5C site identification in an RNA sample and classifying it as a false positive. Likewise, the relaxation of stringency also leads to the likelihood of an abnormal rise in other non-m5C sites being tagged.

In this regard, TP stands for the genuine m5C sites while TN is the non m5C sites. The term FN means the number of actual m5C sites that are wrongly localized and the term FP refers to non-m5C sites that are wrongly localized into m5C sites. One needs to comprehend that these metrics apply exclusively to the systems managing only one class. In examining the performance of the developed system, *FP* and *FN* rates are critical measurements. The present inaccuracy can result to wrong m5C site identification in an RNA sample and classifying it as a false positive. Likewise, the relaxation of stringency also leads to the likelihood of an abnormal rise in other non-m5C sites being tagged.

### 3.2 Data preprocessing

The collected feature set underwent preprocessing, which included applying standard scaling from the sklearn module. The dataset for the analysis was cleansed and any missing values were handled by standard scaling, before feeding the dataset into the machine learning algorithm.

### 3.3 Independent set testing

Independent set test was also conducted on data samples of both the given datasets, namely DS_01 and DS_02. The results have been discussed in the following tabular form. The following table summarizes the results obtained in the table excluding PE ratios. However, the information derived from independent samples was available for *A. thaliana*. However, for other species such as *M. musculus, S. cerevisiae* and *H. sapiens*, and the samples were first divided in the ratio 80:20. Accompanying the experiment results of bagging and boosting ensemble models based on both datasets, the Accuracy and the Precision values have been provided in Table 2 for DS_01 while in Table 3 for DS_02, respectively.

**Table 2.** Independent set test DS_01.

Techniques		*Homo sapiens*				*Mus musculus*				*Arabidopsis thaliana*			
		***ACC*** (%)	***Sp*** (%)	***Sn*** (%)	***MCC*** (%)	***ACC*** (%)	***Sp*** (%)	***Sn*** (%)	***MCC*** (%)	***ACC*** (%)	***Sp*** (%)	***Sn*** (%)	***MCC*** (%)
Stacked		75	80	69	50	79	88	69	59	67	70	64	35
Bagging	**RF**	70	70	70	40	65	64	67	31	69	76	62	39
	**ETC**	71	73	69	42	66	67	68	33	68	78	59	38
	**DT**	63	68	59	27	63	60	67	27	66	73	69	33
	**BC**	100	100	100	100	76	71	82	54	79	88	69	59
Boosting	**GB**	69	74	65	39	65	67	64	31	68	75	60	37
	**HGB**	68	82	56	39	64	67	61	28	70	77	62	40
	**AB**	67	59	78	37	62	61	64	25	67	74	60	34
	**XGB**	65	68	63	31	65	68	63	31	69	65	64	38

**Table 3.** Independent set test DS_02.

Techniques		*Homo sapiens*				*Mus musculus*				*Saccharomyces cerevisiae*				*Arabidopsis thaliana*			
		***ACC*** (%)	***Sp*** (%)	***Sn*** (%)	***MCC*** (%)	***ACC*** (%)	***Sp*** (%)	***Sn*** (%)	***MCC*** (%)	***ACC*** (%)	***Sp*** (%)	***Sn*** (%)	***MCC*** (%)	***ACC*** (%)	***Sp*** (%)	***Sn*** (%)	***MCC*** (%)
Stacked		67	68	65	33	63	64	62	27	65	63	62	28	68	72	65	37
Bagging	**RF**	77	86	69	55	97	94	98	94	97	97	97	95	73	77	69	46
	**ET**	75	69	81	51	100	100	100	100	98	98	100	97	72	78	66	45
	**DT**	70	81	61	43	87	88	85	74	85	95	76	73	70	77	63	40
	**BC**	100	100	100	100	100	100	100	100	98	100	98	97	72	76	68	44
Boosting	**GB**	91	91	92	83	97	94	99	94	96	97	95	92	72	78	67	46
	**HGB**	77	77	100	87	92	88	95	84	94	93	95	88	73	78	69	47
	**AB**	81	77	85	62	92	94	90	84	90	97	83	81	68	73	63	37
	**XGB**	87	88	85	74	84	94	76	71	83	93	73	68	73	78	67	47

**Figure 5.**
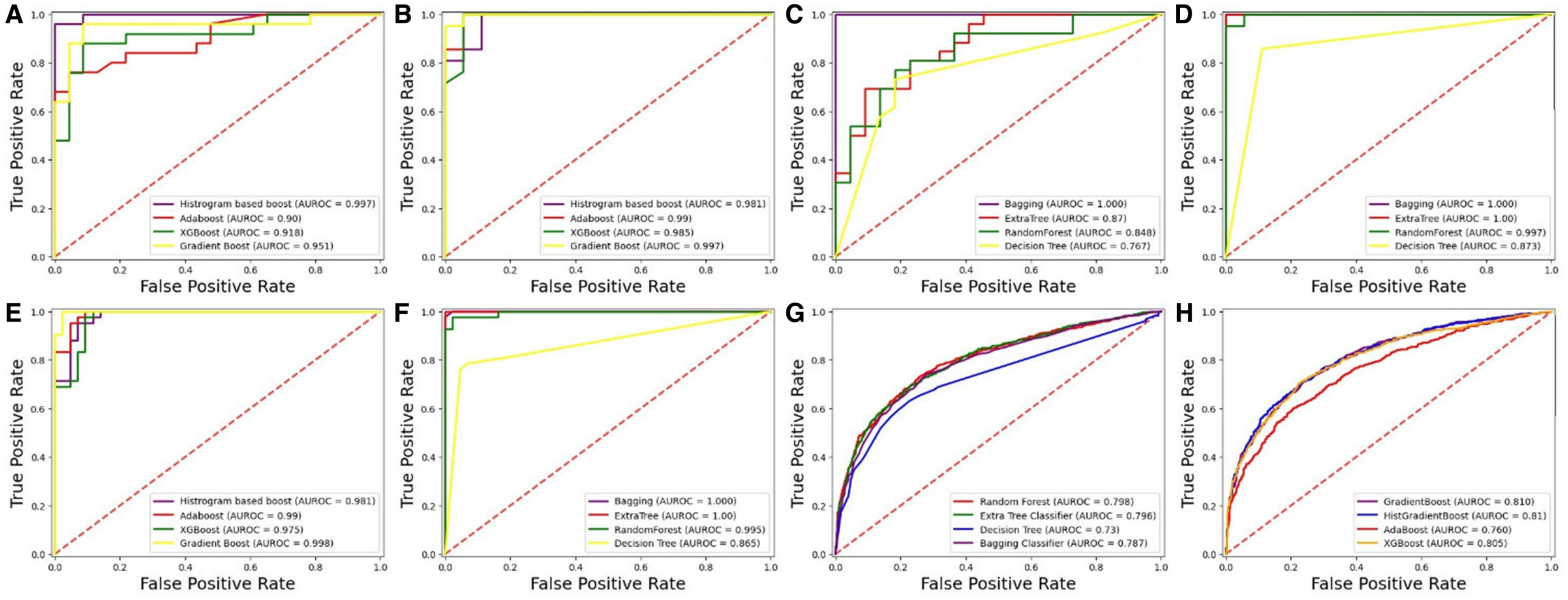
Independent testing ROC graph for DS_01. (A) *Homo sapiens* boosting. (B) *Mus Musculus* boosting. (C) *Homo sapiens* bagging. (D) *Mus Musculus* bagging. (E) *Saccharomyces cerevisiae* boosting. (F) *Saccharomyces cerevisiae* bagging. (G) *Arabidopsis thaliana* bagging. (H) *Arabidopsis thaliana* boosting.

**Figure 6.**
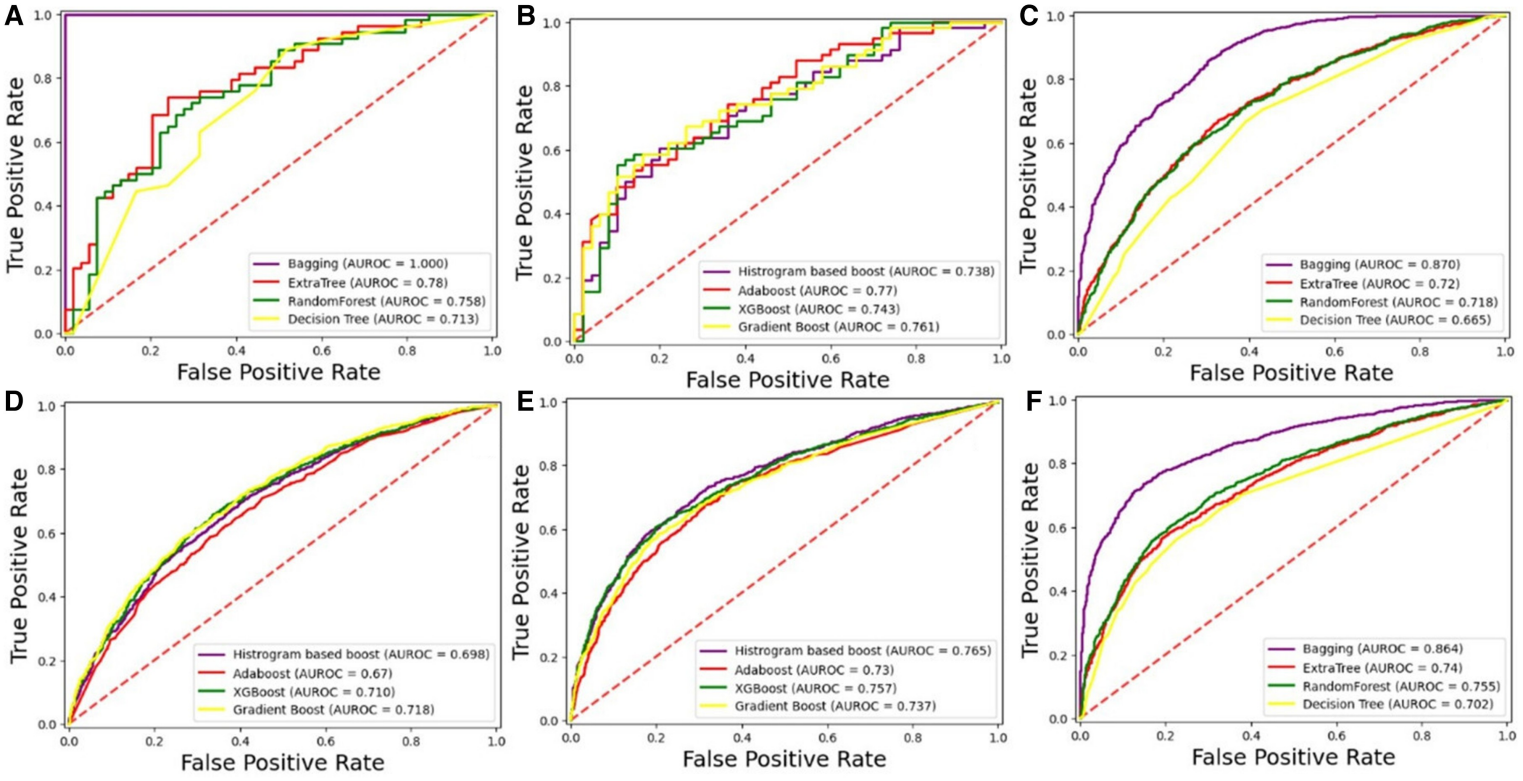
Independent testing ROC graph for DS_02. (A) *Homo sapiens* bagging. (**B**) *Homo sapiens* boosting. (C) *Mus musculus* bagging. (D) *Mus musculus* boosting. (E) *Arabidopsis thaliana* boosting. (F) *Arabidopsis thaliana* bagging.

A receiver operating characteristic (ROC) curve is a graph used to assess the performance of a binary classification model. Figures 5 and 6 present the ROC graph obtained for the independent set test using DS_01 and DS_02, respectively.

### 3.4 K-fold cross-validation

**Figure 7.**
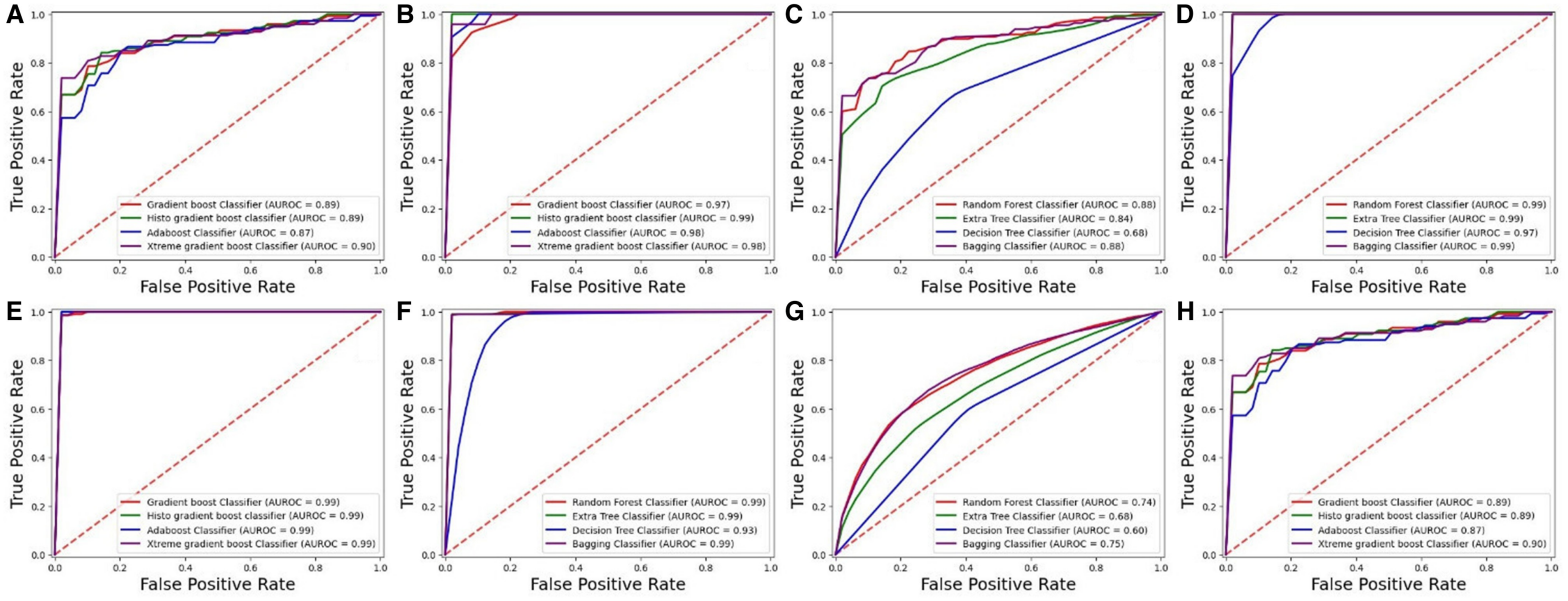
K-Fold testing ROC graph for DS_01. (A) *Homo sapiens* boosting. (B) *Mus musculus* boosting. (C) *Homo sapiens* bagging. (D) *Mus musculus* bagging. (E) *Saccharomyces cerevisiae* boosting. (F) *Saccharomyces cerevisiae* bagging. (G) *Arabidopsis thaliana* bagging. (H) *Arabidopsis thaliana* boosting.

**Figure 8.**
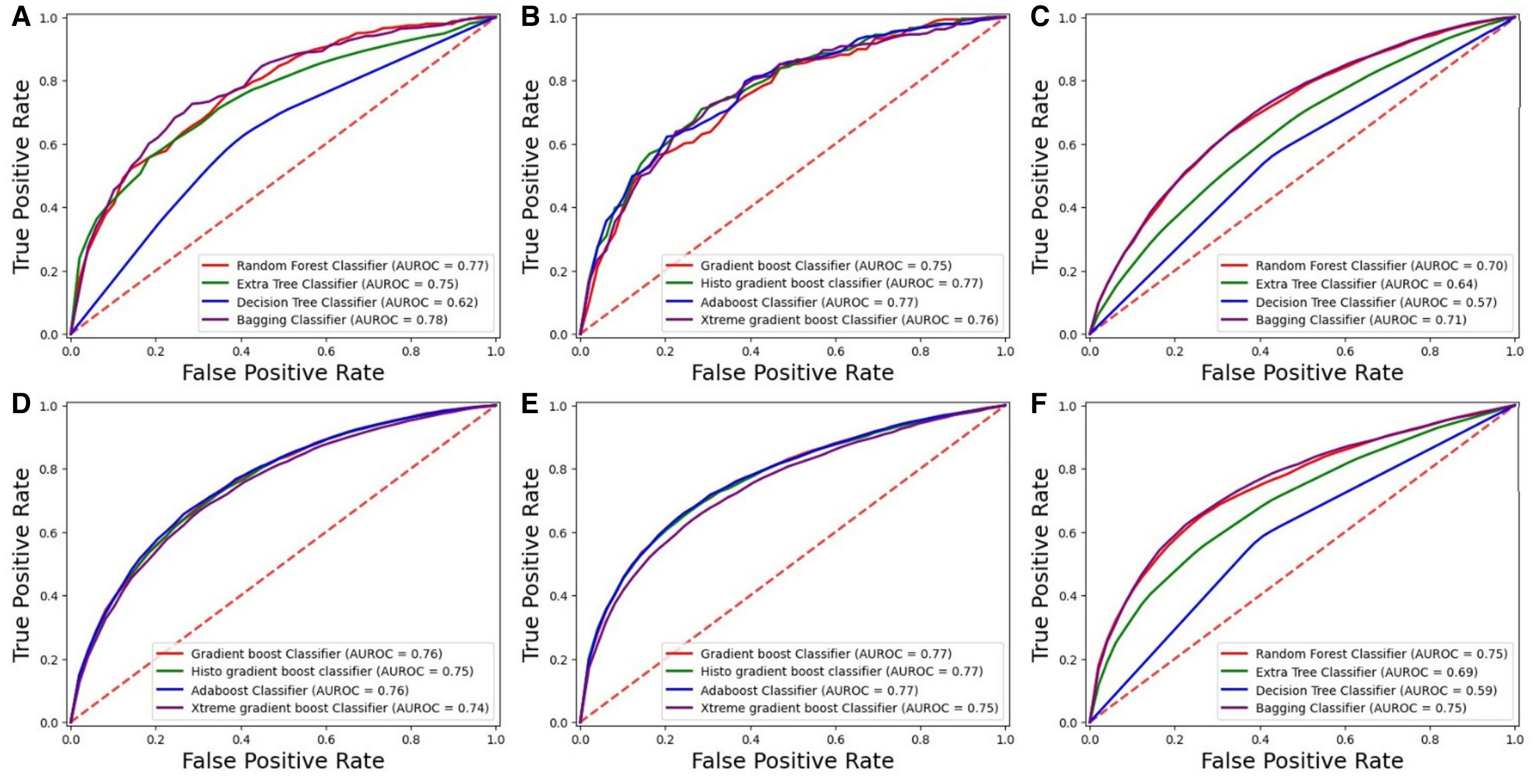
K-Fold testing ROC graph for DS_02. (A) *Homo sapiens* bagging. (B) *Homo sapiens* boosting. (C) *Mus musculus* bagging. (D) *Mus musculus* boosting. (E) *Arabidopsis thaliana* boosting. (F) *Arabidopsis thaliana* bagging.

The cross-validation technique offers a robust and effective method for assessing models using the entire dataset. It divides the data into k subsets, or folds, where each fold serves as a test set while the remaining k-1 folds are used for training. This study set k to 10, so the dataset was split into 10 folds. Each cycle of cross-validation involves training on nine of these folds and testing on the remaining one. This procedure is repeated 10 times, comprehensively evaluating the model’s performance. The cross-validation results are detailed in Tables 4 and 5, which present the performance metrics for each fold. This method helps evaluate the model’s effectiveness across various data segments and assesses its stability. Furthermore, cross validation curves of the ROCs have been represented in Figs 7 and 8 for DS_01 and DS_02, respectively.

**Table 4.** K-Fold Cross-validation results for DS_01.

*Techniques*		*Homo sapiens*				*Mus musculus*				*Saccharomyces cerevisiae*				*Arabidopsis thaliana*			
		*ACC* (%)	*Sp* (%)	*Sn* (%)	*MCC* (%)	*ACC* (%)	*Sp* (%)	*Sn* (%)	*MCC* (%)	*ACC* (%)	*Sp* (%)	*Sn* (%)	*MCC* (%)	*ACC* (%)	*Sp* (%)	*Sn* (%)	*MCC* (%)
Bagging	**RF**	83	73	99	71	89	91	92	74	95	96	96	90	71	69	68	75
	**ETC**	83	99	95	67	98	99	98	97	97	94	99	95	66	65	66	70
	**DT**	87	81	95	75	96	97	98	100	92	82	99	85	58	55	53	62
	**BC**	79	95	75	59	92	93	94	97	97	100	95	95	68	60	62	69
Boosting	**GB**	79	69	95	60	84	77	90	68	98	99	99	99	85	80	81	84
	**HGB**	75	78	70	48	100	100	100	100	95	96	98	90	85	79	80	84
	**AB**	75	95	61	53	100	100	100	100	100	100	100	100	84	78	79	82
	**XGB**	91	95	95	83	100	100	100	100	97	95	99	95	88	84	85	88

**Table 5.** K-Fold cross-validation results for DS_02.

*Techniques*		*Homo sapiens*				*Mus musculus*				*Arabidopsis thaliana*			
		*ACC* (%)	*Sp* (%)	*Sn* (%)	*MCC* (%)	*ACC* (%)	*Sp* (%)	*Sn* (%)	*MCC* (%)	*ACC* (%)	*Sp* (%)	*Sn* (%)	*MCC* (%)
Bagging	**BC**	73	76	68	45	70	75	64	40	65	67	64	31
	**RF**	71	72	70	42	69	75	63	39	65	67	63	30
	**ETC**	69	74	63	38	64	74	55	29	59	68	50	19
	**DT**	62	61	63	24	59	59	58	18	57	57	56	13
Boosting	**XGB**	72	69	74	44	68	72	65	37	68	68	68	36
	**AB**	71	70	70	41	70	77	63	41	69	70	69	38
	**GB**	70	70	70	40	70	77	64	41	69	69	69	38
	**HGB**	70	70	71	41	70	75	65	41	69	69	69	38

**Figure 9.**
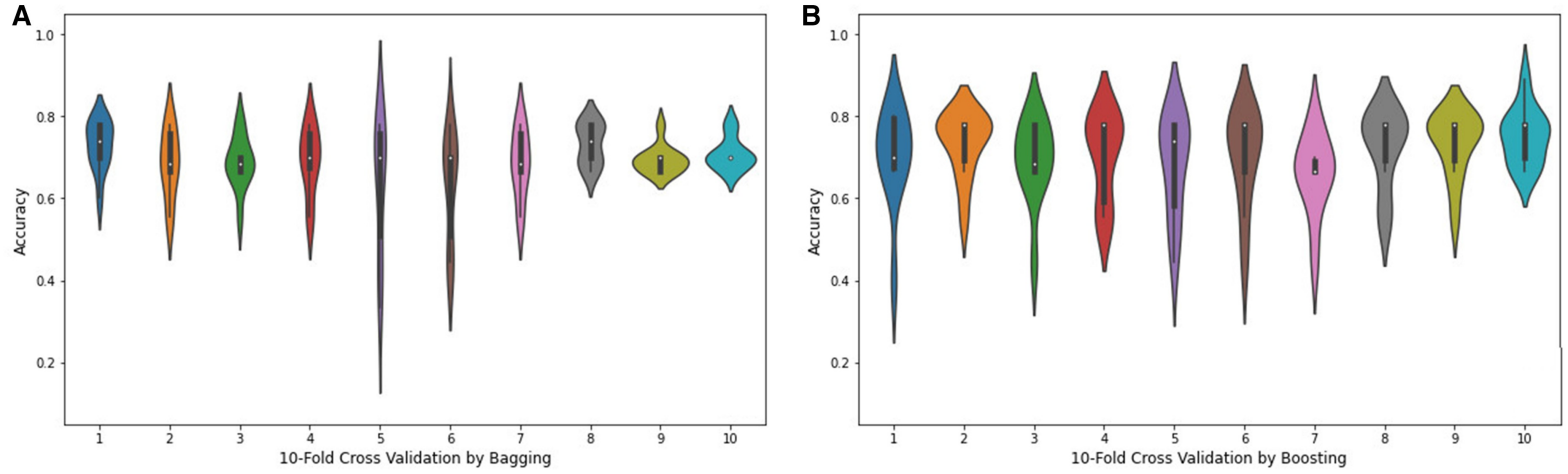
Violin plots of the accuracy values obtained from 10-fold cross validation through (A) bagging and (B) boosting.

**Figure 10.**
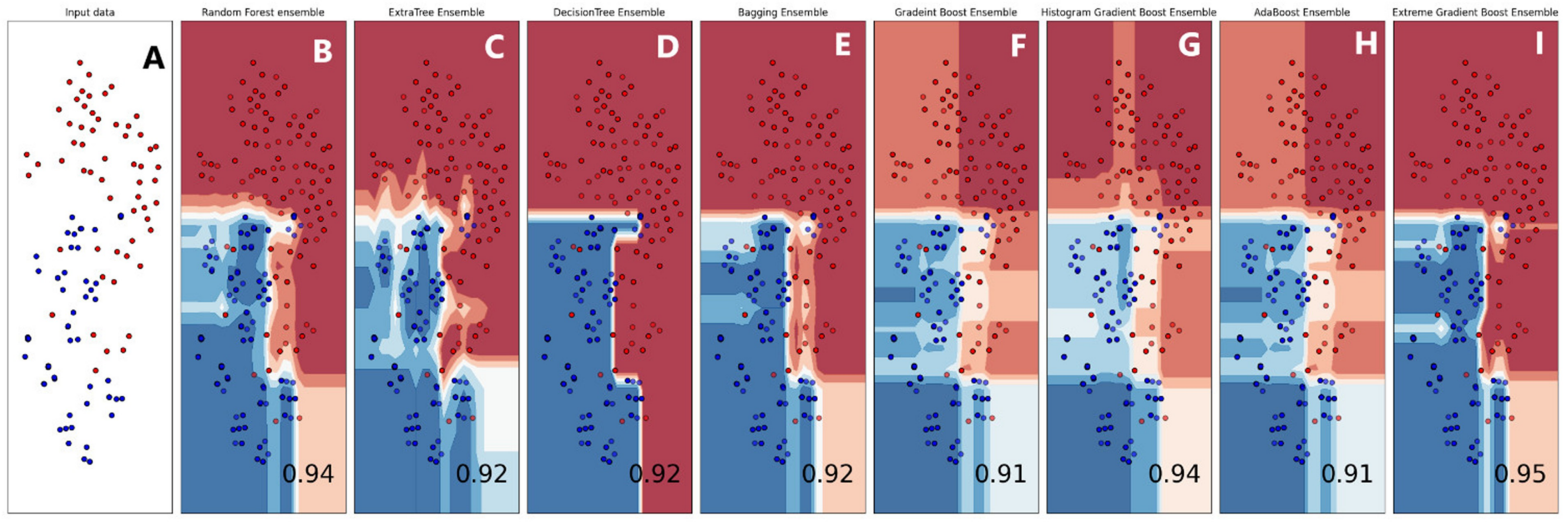
Boundary visualization of Ensemble models. (A) Input. (B) Random Forest. (C) Extratree. (D) DecisionTree. (E) Bagging. (F) Gradient Boost. (G) Histogram Boost. (H) Adaboost. (I) Extreme Gradient Boost.

The violin plot is a graphical method for displaying numerical data across one or more groups using density curves. In these plots, the white dot indicates the median, the black bar shows the interquartile range, and the two dark lines extending from the bar represent the lower and upper adjacent values. Violin plots are shown in Fig. 9 to represent the accuracy values of the folds of the highest achieving bagging and boosting ensemble models. Supervised machine learning models related to classification issues can be useful indeed, but sometimes the typical numeric predictions are insufficient. It is essential to envisage the actual decision boundary that splits different groups. Thus, in this study, it was decided to subject the classification algorithms to a decision surface analysis to increase classification accuracy. In the current decision surface map, the outcome over the map across the input feature space is determined by the response of the trained machine learning model. Before applying the model, it was first trained on the training dataset; afterwards, the trained model was used to predict a set of input space values. To plot, contour_f() function of matplotlib was used and for scatter plot. As applied in this study, classification algorithms’ decision surface plots are depicted in the Fig. 10.

### 3.5 Comparison with the existing predictors

The deep learning models in this study were trained and tested using two separate datasets. In said evaluation, we compared the proposed model with previous research against iRNA-m5C and m5c-pred-XS. We taken out *A. thaliana* holding samples to best match and independent screening between ZY-m5C and probable nonsense with iRNA-m5C. Also for independent testing, the standard train-test split method was implemented when comparing with m5C-pred-XS. We compared the results from 10-fold cross-validation tests. Table 6 presents the results, with the bold values indicating the outcomes of the proposed model in comparison to the state-of-the-art methods.

**Table 6.** Comparative analysis of m5c-iDeep with other m5C site predictors.

	*Homo sapiens*				*Mus musculus*				*Saccharomyces cervisiaie*				*Arabidopsis thaliana*			
	Acc (%)	Sp (%)	Sn (%)	MCC	Acc (%)	Sp (%)	Sn (%)	MCC	Acc (%)	Sp (%)	Sn (%)	MCC	Acc (%)	Sp (%)	Sn (%)	MCC
**iRNA-m5c** [**14]**	90.8	91.7	90.0	0.81	100	100	100	1.00	100	100	100	1.00	70.7	75.7	65.7	0.41
**M5C-pred XS** [**12]**	80.4	89.9	71.0	0.62	72.3	85	59.5	0.46					77.2	80	74.4	0.53
**m5C-iEnsem**	**100**	**100**	**100**	**1.00**	**100**	**100**	**100**	**1.00**	**96**	**97**	**95**	**0.92**	**79**	**88**	**69**	**0.59**

The bold values indicating the outcomes of the proposed model in comparison to the state-of-the-art methods.

The study presented a powerful model for detecting m5C sites, using a new feature extraction technique and advancing deep learning models’ development, training, and evaluation. Cytosine methylation at the 5-position in RNA, facilitated by specific enzymes, is believed to contribute to various biological processes, including gene expression regulation, RNA splicing, and mRNA stability. This modification has also been linked to diseases such as cancer and intellectual disability syndromes. The results showed that m5C-iEnsem outperformed other models in both 10-fold cross-validation and independent testing. The m5C-iEnsem model enhances RNA 5-methylcytosine (m5C) site prediction by leveraging ensemble learning for improved accuracy and generalization. It integrates both sequence and structural features of RNA, making it more effective in identifying m5C modifications, which play crucial roles in RNA metabolism and disease. This model could become a benchmark for future RNA modification prediction, with applications in biological research and medical fields.

## 4 Web-server accessibility

A web server provides a convenient and efficient platform for users to conduct computational analyses easily. The m5C-iEnsem web server has been developed to support the proposed model, offering free access to facilitate these analyses. Users can visit the web server via this link: https://m5c-iensem.streamlit.app/.

### 4.1 Web-servers exception

**Figure 11.**
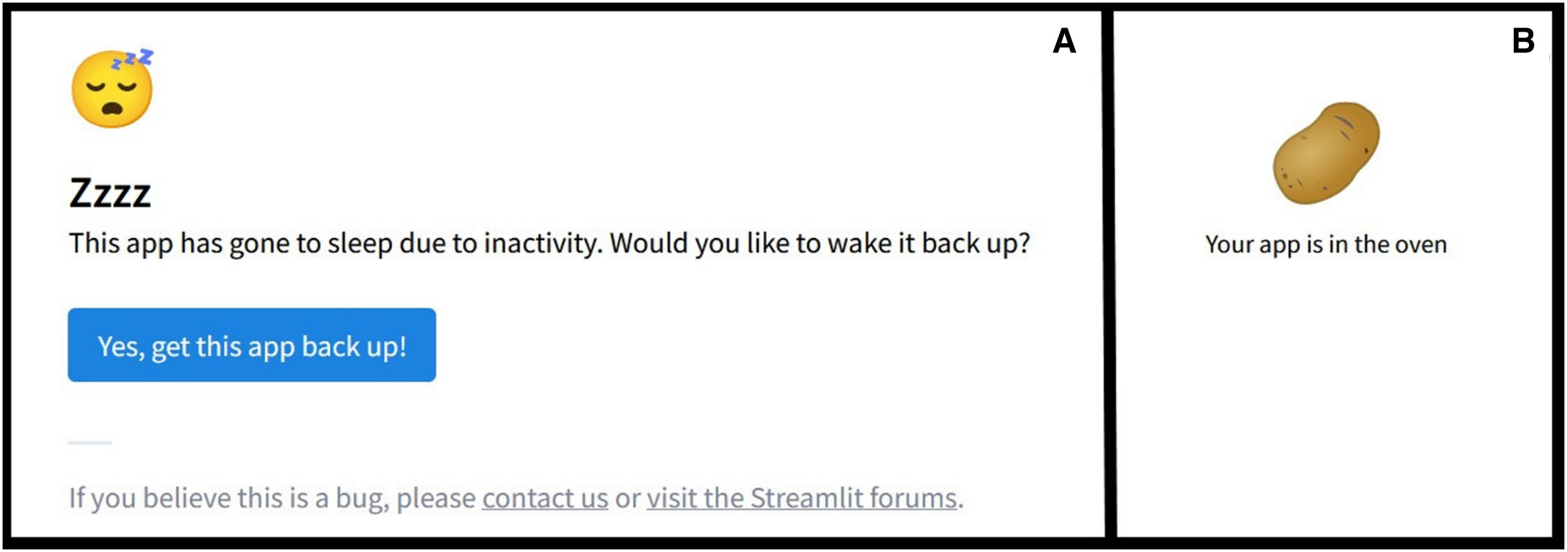
(A) Web-server is in “Hibernate” mode after long inactivity. (B) Re-activation of web-server in progress.

Web servers remain active during regular use but may enter a hibernation state after extended inactivity. A dialogue box will appear when a user accesses the server link during such periods (as illustrated in Fig. 11), indicating that the server is inactive, not deleted. To reactivate the server, the user simply needs to click “Yes, get this app back up!”. After a short wait, the server will be restored. As shown in Fig. 11B, a loading message will be displayed during the re-activation process.

## 5 Conclusion

This manuscript uses Ensemble learning approaches for detecting m5C, one of the most common RNA post-transcriptional modifications. The RNA sequences were thus analyzed using positional and compositional feature extraction techniques of nucleotides. The incorporation of statistical moments helped reduce the dimensionality of features. After that, the acquired dataset was trained through several ensemble learning models, including boosting and bagging. The trained models were further evaluated using cross-validation to estimate their efficiency. An independent set test has been carried out using *A. thaliana*. The effectiveness of the models was determined by means of performance indicators, including specificity, sensitivity, Matthew’s correlation coefficient and accuracy. Finally, the best bagging ensemble model was utilized to build the proposed m5c-iEnsem. For assessing m5c-iEnsem, the latter was compared to other available predictors. From the above analysis, the performance of m5c-iEnsem has the greatest score for the measures of accuracy than other studies. Thus, it can be concluded that the suggested model improves upon the previously mentioned methods for identifying modified m5c sites.

Conflict of interest: None declared.

## Data Availability

The data and code relevant to this research can be accessed via the following link: https://github.com/taseersuleman/m5c-iEnsem.
